# Juvenile idiopathic arthritis: from aetiopathogenesis to therapeutic approaches

**DOI:** 10.1186/s12969-021-00629-8

**Published:** 2021-08-23

**Authors:** Lina N. Zaripova, Angela Midgley, Stephen E. Christmas, Michael W. Beresford, Eileen M. Baildam, Rachel A. Oldershaw

**Affiliations:** 1 Department of Musculoskeletal and Ageing Science, Institute of Life Course and Medical Sciences, University of Liverpool, William Henry Duncan Building, 6 West Derby Street, Liverpool, L7 8TX UK; 2 Department of Women and Children’s Health, Institute of Life Course and Medical Sciences, University of Liverpool, University Department, Liverpool Women’s Hospital, First Floor, Crown Street, Liverpool, L8 7SS UK; 3 Department of Clinical Infection, Microbiology and Immunology, Faculty of Health and Life Sciences, Institute of Infection, Veterinary and Ecological Sciences, University of Liverpool, The Ronald Ross Building, 8 West Derby Street, Liverpool, L69 7BE UK; 4 Department of Paediatric Rheumatology, Alder Hey Children’s NHS Foundation Trust, East Prescott Road, Liverpool, L14 5AB UK

**Keywords:** Juvenile idiopathic arthritis, Pathogenesis of juvenile idiopathic arthritis, Aetiology of juvenile idiopathic arthritis, Disease-modifying anti-rheumatic drug treatment

## Abstract

Juvenile idiopathic arthritis (JIA) is the most common paediatric rheumatological disorder and is classified by subtype according to International League of Associations for Rheumatology criteria. Depending on the number of joints affected, presence of extra-articular manifestations, systemic symptoms, serology and genetic factors, JIA is divided into oligoarticular, polyarticular, systemic, psoriatic, enthesitis-related and undifferentiated arthritis. This review provides an overview of advances in understanding of JIA pathogenesis focusing on aetiology, histopathology, immunological changes associated with disease activity, and best treatment options. Greater understanding of JIA as a collective of complex inflammatory diseases is discussed within the context of therapeutic interventions, including traditional non-biologic and up-to-date biologic disease-modifying anti-rheumatic drugs. Whilst the advent of advanced therapeutics has improved clinical outcomes, a considerable number of patients remain unresponsive to treatment, emphasising the need for further understanding of disease progression and remission to support stratification of patients to treatment pathways.

## Introduction and classification

Juvenile idiopathic arthritis (JIA) unifies all forms of chronic childhood arthritis, affecting not only joints, but extra-articular structures, including eyes, skin, and internal organs, leading to disability and even associated fatality. It is defined as the presence of arthritis of unknown aetiology that begins before the age of 16 and persists for at least 6 weeks [1].

The International League of Associations for Rheumatology (ILAR) stratifies subtype of autoimmune inflammatory disorders, determined by the number of joints affected, the presence of systemic symptoms and detection of rheumatoid factor (RF). JIA is divided into the sub-forms: oligoarticular (persistent or extended), polyarticular (RF-negative or RF-positive), systemic (sJIA), psoriatic arthritis and enthesitis-related arthritis, with each differing in genetic susceptibility and severity of arthritis [1]. Any arthritis that does not fit into these categories or corresponds to > 1 subtype is considered as undifferentiated [2]. The main characteristics of arthritis, internal organ involvement, genetic predisposition, laboratory markers of each subtype and adult equivalent are shown in Table 1.

**Table 1:** The main clinical and laboratorial characteristics of JIA subtypes based on International League of Associations for Rheumatology (ILAR) classification criteria. ANA – antinuclear antibodies, Anti-CCP – Anti-Cyclic Citrullinated Peptide, CRP - C-reactive protein, ERA - enthesitis-related arthritis, HLA - human leukocyte antigen system, RF – rheumatoid factor, sJIA – systemic JIA

Subtype	Oligoarticular JIA	Polyarticular JIA RF-	Polyarticular JIA RF+	ERA	Psoriatic JIA	Systemic sJIA
Characteristic of arthritis	• ≤4 joints affected• Mainly large joints• Asymmetric, often only a single joint (knee)	• ≥5 joints affected• Symmetric or asymmetric• Small and large joints• Sometimes a cervical spine and/or temporomandibular joint	• ≥5 joints affected• Symmetric• Mainly small joints (metacarpophalangeal joints and wrists)• Erosive• Aggressive symmetric polyarthritis	• Lower limb joints affected more common• Axial involvement: sacroiliac joint, hip or shoulder	• Asymmetric arthritis• Small and large joints	• Usually arthralgias;• 30–50% chronic arthritis - slowly developed• Mostly wrists, knees, ankles joints or asymptomatic temporomandibular arthritis
Systemic manifestation	30% uveitis	10% uveitis	• Rheumatoid nodules• 10% uveitis	• Acute anterior uveitis• EnthesitisGut inflammation	• Psoriasis• Dactylitis• Onycholysis• Nail pitting• Uveitis (10–15%)	• Spiking fever• Generalized lymphadenopathy• Migratory salmon-pink rash• Serositis (pericarditis most common, then pleuritic and peritonitis)• Hepatosplenomegaly• MAS
Sex predominance	Female	Female	Female	Male	Equal	Equal
HLA genetic pre-disposition	Associated with• *A2*• DRB1*11• DRB1*08• DPB1*0201• DRB1*15*01• DQA1*04• DQB1*04• DRB1*13 for persistent• DRB1*01 for extended variant	Associated with• *A2*• DRB1*08• *DPB1:03*• DQA1*04• DRB1*15*01• DPB1*02*01	Associated with• DRB1*04• DRB1*01• DRB1*08• DQA1*03	Associated with• *B27*• DRB1*01• DQA1*01• DQB1*05	Associated with• DRB1*01• DRB1*11• DRB1*12• HLA-C*06 - biomarker for skin involvement• *B27* – for sacroileitis (mostly in older age)	Associated with• *DRB1:04*• DQA1*01• DQB1*04• DRB1*01
Biomarkers	60% ANA+	40% ANA+	• RF+• Anti-CCP+• ANA+ in 40%	45–85% *HLA-B27*+	50% ANA+	Elevating level of• CRP• Ferritin• Platelets
Adult equivalent	–	Potential Seronegative Rheumatoid Arthritis	RF-positive Rheumatoid Arthritis	Spondyloarthropathies	Psoriatic Arthritis	Adult Onset Still’s Disease

Oligoarticular JIA is characterised by inflammation of up to four joints that archetypically proceeds as asymmetrical arthritis predominantly affecting the joints of the lower extremities, such as knee and ankle, with high frequency of positivity to anti-nuclear antibody (ANA) and high risk of chronic uveitis [3]. Polyarticular JIA (pJIA) affects five or more large/ small joints and is hallmarked by injury to the metacarpophalangeal joints and wrists [4]. Both RF-positive and negative variants have characteristic clinical features. RF-negative pJIA, inflammation can be asymmetrical, but for RF-positive pJIA symmetric involvement of the large and small joints of hands and feet is the most prevalent. Enthesitis-related arthritis (ERA) resembles oligoarthritis, affecting the joints of the lower limb in association with enthesitis. Due to the association with lower limb and sacroiliac joints, enthesitis, uveitis and the association with HLA-B27, Ravelli et al. (2007) have suggested ERA to be a disease belonging to the group of spondyloarthropathies [1]. Psoriatic arthritis often proceeds as oligoarthritis or RF-negative polyarthritis and involves more commonly the small joints accompanied by dactylitis, psoriatic rash and/ or nail pitting [1, 3]. Psoriatic JIA itself is described as a heterogeneous disease where children < 6 years are more likely to be female, ANA-positive and predisposed to chronic uveitis, with arthritis of wrists and small joints of the hands and feet. In older children disease is associated with HLA-B27 positivity, enthesitis and axial disease with male predisposition [5].

Standing apart from other subtypes, sJIA manifests not only with widespread joint arthritis, but also with a significant range of systemic inflammation symptoms (Table 1) [6]. Approximately 10% of sJIA patients present systemic symptoms with associated macrophage activation syndrome (MAS), a potentially life-threatening condition with histopathological features that include the accumulation of terminally-differentiated macrophages with high hemophagocytic activity [7, 8]. Currently the limitations of the ILAR classification scheme are pertinent and include the absence of link to pathogenesis, molecular pathways and response to the therapy [9]. In addition, there is a substantial unclassified cohort of patients with JIA onset before 6 years of age and female predominance having specific features that include symmetric arthritis, iridocyclitis, ANA and HLA-DR8-positivity. In 2019 the Pediatric Rheumatology International Trials Organization (PRINTO) Consensus revised the ILAR classification criteria and proposed to identify this complex of features as early-onset ANA-positive JIA [10]. Other JIA disorders identified according to these preliminary criteria include sJIA, RF-positive JIA and enthesitis/spondylitis-related JIA (Table 2). Arthritis of more than 6 weeks duration that does not fit the criteria is grouped as ‘Other JIA’, and that fitting more than one criterion, ‘unclassified JIA’ [10]. PRINTO Consensus highlights JIA as not a single disease but a group of different disorders, of which diagnosis does not require joint count or the presence of arthritis. The onset of the disease has been changed to before 18 years of age [10].

**Table 2:** The preliminary criteria of the main JIA disorders proposed by the Pediatric Rheumatology International Trials Organization (PRINTO) Consensus. The PRINTO classification proposed systemic JIA (sJIA), rheumatoid factor–positive JIA (RF+ JIA), enthesitis/spondylitis-related JIA and early-onset antinuclear antibody–positive (early-onset ANA+) JIA. ANA – antinuclear antibodies, AOSD - adult-onset Still’s disease, Anti-CCP – Anti-Cyclic Citrullinated Peptide, HLA - human leukocyte antigen system, RA – rheumatoid arthritis, RF – rheumatoid factor, y.o. – years old

JIA disorders	Early-onset ANA+ JIA	RF+ JIA	Enthesitis/ spondylitis- related JIA	sJIA
Clinical criteria	• Arthritis for ≥6 weeks, and • Early-onset (≤ 6 y.o.)	• Arthritis for ≥6 weeks	• Peripheral arthritis and enthesitis, or • Arthritis or enthesitis, + ≥ 3 months of sacroiliitis, or • Arthritis or enthesitis + 2 of the following: - sacroiliac joint tenderness - inflammatory back pain - acute anterior uveitis - history of a SpA in relative	• Fever with exclusion of infectious, neoplastic, autoimmune, or monogenic autoinflammatory diseases, for at least 3 days and reoccurring over 2 weeks + 2 major criteria or 1 major criterion and 2 minor criteria **Major criteria**: • evanescent erythematous rash • arthritis **Minor criteria**: - generalized lymphadenopathy and/or hepatomegaly and/or splenomegaly - serositis - arthralgia lasting more than 2 weeks
Laboratory criteria	• 2 “+” ANA tests with a titer ≥1/160 at least 3 months apart	• 2 positive tests for RF at least 3 months apart or • 1 positive test for anti-CCP	- presence of HLA-B27 antigen	- leukocytosis (≥ 15,000/mm3) with neutrophilia
Adult equivalent	–	RF-positive RA	Spondyloarthritis	AOSD

## Histopathology of JIA

**Fig. 1:**
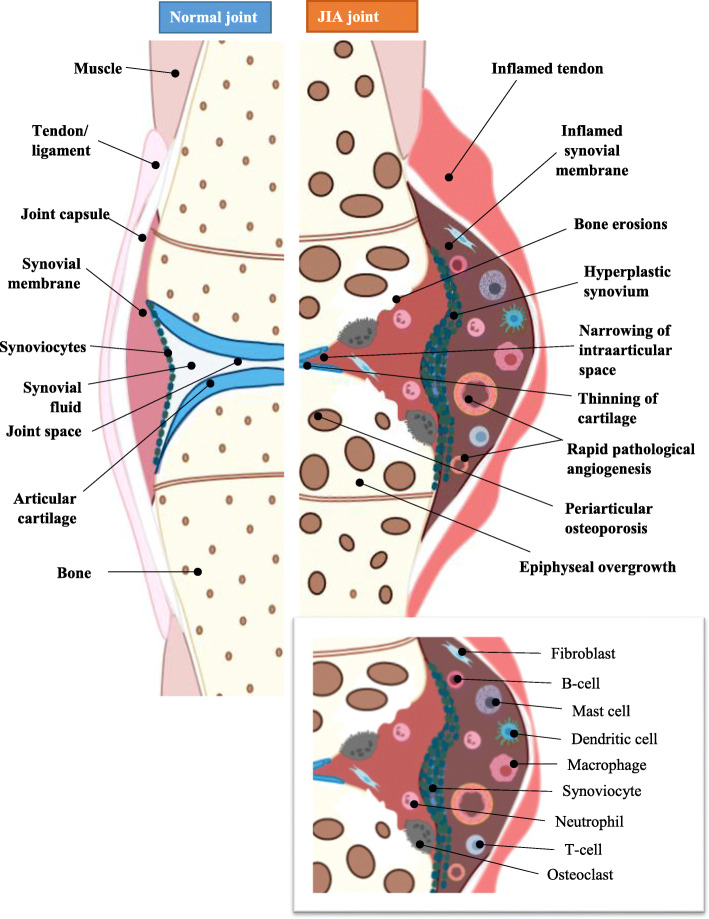
Schematic diagram showing the differences between the normal and JIA joint. The pathological process within the JIA synovial joint is characterised by uncontrolled proliferation of synoviocytes resulting in increased number of layers and thickening of the synovial membrane; rapid pathological angiogenesis; formation of pathological synovium, “pannus”, with uncontrolled growth and invasive properties; accumulation of granulocytes, macrophages, plasma cells, lymphocytes and the production of inflammatory mediators, provoking synovitis. Created with BioRender.com

The main hallmark of JIA is joint inflammation with tissue destruction [11]. Within the synovial joint, the synovial membrane thickens in response to uncontrolled proliferation of synoviocytes and immunocompetent cells, including T-cells, B-cells, natural killers, neutrophils, macrophages, dendritic cells and plasma cells that infiltrate the sub-lining layer of the synovium (Fig. 1) [11].

Hyperplasia and hypertrophy of the synovium causes intraarticular hypoxia, increasing the production of pro-angiogenic mediators and initiating pathological angiogenesis [12]. Increased concentrations of vascular endothelial growth factor (VEGF), a potent endothelial cell (EC) mitogen, its soluble receptors-1 and -2 (sVEGF-R1, sVEGF-R2), and osteopontin (OPN), a chemotactic factor that activates mononuclear cells, have been correlated to synovial angiogenesis assessed by Doppler ultrasonography of JIA patients [12]. Angiopoietin-1 (Ang-1), another pro-angiogenic EC mitogen with a role in stabilisation of newly formed vessels was also shown to be upregulated in JIA. New blood vessel formation within the synovium increases blood supply and the migration of pro-inflammatory cells into the joint, forming a pathological synovium, known as ‘pannus’ (Fig. 1).

Granulocytes, macrophages, plasma cells and lymphocytes accumulate in the subintima of the joint and produce pro-inflammatory mediators including tumour necrosis factor-α (TNFα) and interleukin (IL)1β, upregulating pannus-synoviocyte production of catabolic proteases including matrix metalloproteinases (MMPs, particularly MMP1 and MMP3), aggrecanases and cathepsins, that breakdown the extracellular matrix of the articular cartilage tissue causing loss of function, biomechanical strength and the ability to smoothly articulate the joint [2, 13]. Pro-inflammatory cytokine-mediated activation of receptor activator of nuclear factor-kappaB (RANK)-expressing osteoclasts results in bone erosion [14]. Damage to cartilage and bone in the advanced stages of JIA causes ankyloses and the loss of movement in the affected joints. Considering that JIA is a disease of the developing body, patients with JIA are likely to suffer from disruption of skeletal growth [15].

## Aetiology

The heterogeneity of JIA disease subtypes adds complexity to the investigation of cause and mechanism of pathogenesis, and the initiating factors of JIA remain unresolved [16].

Environmental factors, including infectious agents, vaccinations, antibiotics, vitamin D deficiency, stress and trauma have been proposed as risk factors. Infectious viruses (*Epstein-Barr virus*, *Parvovirus B*, *Rubivirus*, *Hepatitis B virus)* and bacteria (*Salmonella spp*., *Shigella spp*., *Campylobacter spp*., *S. pyogenes, B. henselae, M. pneumoniae*, *Chlamydophila pneumonia)* have been reported as causal factors provoking JIA [17]*.* Gastrointestinal infection leading to loss of gut microbiome diversity and disrupted tryptophan metabolism increases the risk of ERA [18]. Carlens et al. (2009) reported that maternal smoking during pregnancy increased the probability of an immune imbalance during foetal development leading to the onset and progression of paediatric arthritis [19]. In contrast, some beneficial factors such as breast feeding and household siblings might decrease the risk of developing JIA [20].

Several studies have documented genetic associations to JIA [21–25]. Genetic linkage depends on subtype and may be divided into two groups: HLA genes and non-HLA-related genes. Meta-analysis of genetic predisposition to JIA subtypes has shown association with HLA class II molecules (*A2, DRB1, DPB1*) mostly for non-systemic subtypes (Table 1), while for sJIA the lack of association with HLA genes has been found [21]. Oligoarticular JIA is associated with A2, DRB1*11, DRB1*08, DPB1*02, DRB1*13, DRB1*15*01 and DRB1*01, while for* RF- *polyarticular the most commonly associated genes are *DPB1:03* and *DRB1:08, *and for RF+ JIA, DRB1*04 and DRB1*01 [23]*.* Of interest, HLA-A, HLA-B and HLA-DR were observed in females with oligoarthritis but not males, which may point to disease heterogeneity [2]. The main gene associated with ERA is *HLA-B27*, with other genes predisposing the development of ERA being DRB1*01, DQA1*01, and DQB1*05 [18]. *HLA-B27* is also found in late-onset psoriatic JIA [5].Genetic pre-disposition of non-HLA-related genes plays a pivotal role in the onset of inflammatory response leading to tissue damage. Genes encoding cytokines TNF, IL2, IL10, IL6, macrophage migration inhibitory factor (MIF), protein tyrosine phosphatase (PTPN22), signal transducer and activator of transcription-4 (STAT4), solute carrier family-11 (proton-coupled divalent metal ion transporters), member-1 (SLC11A1), natural resistance-associated macrophage protein-1 (NRAMP1) and WNT1-inducible signalling pathway protein-3 (WISP3) have all been associated with JIA [2, 21, 23]. Polymorphism in genes encoding endoplasmic reticulum resident aminopeptidases (*ERAP1* and *ERAP2*) predispose to ERA, while genes encoding IL1, IL6, IL10 and MIF increase the risk of sJIA, which itself is considered as a genetically distinct subtype of JIA [16].

## Pathogenesis of JIA

JIA subtypes represent a heterogeneous group of diseases with multifactorial and different pathogenesis (Table 3). It is not completely understood how the combination of the environmental triggers and genetic susceptibility disrupt the balance between regulatory and effector cells in the pathogenesis of JIA.

**Table 3:** Differences in the pathogenesis between oligoarticular, polyarticular rheumatoid factor (RF)-negative and positive, systemic (sJIA), psoriatic arthritis and enthesitis-related arthritis (ERA). As an autoinflammatory disease sJIA is different in pathogenesis, clinical manifestations, and therapeutic strategy compared to non-systemic subtypes of JIA. ANA - Antinuclear antibodies, anti-MCV - antibodies against mutated citrullinated vimentin, anti-CCP - anti-cyclic citrullinated peptide, IL* -* interleukin, MIF - macrophage migration inhibitory factor, PsJIA – psoriatic JIA, RF – rheumatoid factor, sJIA – systemic JIA, TNF - tumour necrosis factor

	Oligoarticular JIA	Polyarticular JIA	ERA	Psoriatic JIA	sJIA
Type of disease	Autoimmune	Autoimmune	Autoimmune	Early-onset PsJIA – autoimmune, while late-onset PsJIA – autoinflammatory	Autoinflammatory
Immune system mainly involved in pathogenesis	Adaptive immune system	Adaptive immune system	Adaptive immune system	Adaptive immune system in early-onset PsJIA Innate immune response in late-onset PsJIA	Innate immune system
Gene association	MHC class II	MHC class II	*HLA-B27*	*HLA-B/C, HLAB, IL12B, IL23R, TNP1, TRAF3IP3, REL* *HLA-B27* in late-onset PsJIA	*TNF, IL6, IL10, MIF, IL1*
Antibodies	ANA	ANA RF, anti-CCP, anti-MCV – for RF+ JIA	ANA may be positive in some cases	ANA in the early-onset PsJIA	–
Predominant effector cells	CD4^+^, CD8^+^ T-cells, neutrophils	CD4^+^, CD8^+^ T-cells	γδT-cells, Th17 cells	Th1 and Th17 cells subsets, macrophages	Monocytes, macrophages, neutrophils
Key moment in pathogenesis	Imbalance between inflammatoryTh1/Th17 and Treg cells	Imbalance between pro-inflammatory Th1/Th17 and Treg cells	HLA-B27 involved in presentation of unidentified arthritogenic peptide caused T-cells activation and induction of endoplasmic reticulum stress	Autoinflammatory activation at the synovial-entheseal complex Autoimmune processes in extra-articular tissues	Abnormal activation of phagocytes leads to hypersecretion of pro-inflammatory cytokines
Main pro-inflammatory cytokines	TNFα, IL17, IFNγ	TNFα, IL17, IL33, IFNγ	TNFα, IL17, IL23	IL17, IL23	IL1, IL6, IL18, IL37, LRG and ADA2
Main treatment targets	Inhibition of T-cell proliferation, rarely anti-TNFα therapy is needed	Inhibition of T-cell proliferation, block of TNFα	Block of TNFα	Inhibition of T-cell proliferation, block of TNFα	Block of IL1 and IL6 signalling pathway

### Immunological changes

Initiation of the JIA pathophysiological cascade includes abnormal activation of T-cells, B-cells, natural killer (NK) cells, dendritic cells (DC), macrophages and neutrophils and the production of pro-inflammatory mediators that cause joint destruction and systemic complications.

Oligoarticular and pJIA are characterised by autoreactive antigen-specific T-cells and high titres of autoantibodies, and typically shows strong associations with MHC class II alleles. Breakdown of immunologic self-tolerance involves MHC class II alleles suggesting a pivotal role for CD4+ T helper (Th) cells [21]. Inflammation is considered to be a consequence of disrupted balance between pro-inflammatory Th1/Th17 and anti-inflammatory regulatory T-cells (Treg). The decrease in Treg cells is inversely correlated with an increase in the Th17 cell population and occurs from the differentiation of naïve T-cells by influence of IL1β [26]. Differentiation of naive T-cells into Th-cells results in the production of pro-inflammatory cytokine IL17, which may induce production of IL6, MMP1 and 3, IL8 (a chemoattractant for neutrophils) by synoviocytes, resulting in subsequent joint destruction [16, 26]. Prelog et al. (2008) revealed premature immunosenescence of T-cells in oligoarticular, pJIA and sJIA patients, as indicated by the loss of compensatory proliferation of naive T-cells, increased telomeric erosion, and loss of capability of the thymus to produce T-cell receptor excision circles [27].

Association with HLA class II and the presence of ANA suggests that an adaptive immune response is predominant in the pathogenesis of oligoarticular JIA. However, activated neutrophils with altered phenotype and dysfunction, and impaired synovial monocytes and macrophages with reduced capacity to phagocytose have recently been identified in synovial fluid of patients with oligoarticular JIA [28, 29]. Together with high levels of monocyte-derived cytokines this emphasises the importance of the innate immune system in oligoarticular JIA pathogenesis [28, 29].

The pathogenesis of ERA is driven by HLA-B27-mediated presentation of arthritogenic peptide following T-cell activation and IL23 and IL17 secretion. Bowel wall inflammation usually accompanied with ERA is driven by γδT cells, innate lymphoid cells type-3 or Th17 cells and IL17 and IL23 production [18]. Enthesitis is triggered by repeated biomechanical stress stimulation resulting in microtrauma and release of fibronectin, hyaluronan, and other molecular components from damaged connective tissue, which may directly activate synovial macrophages, stromal cells and IL23 production to establish a positive feedback loop. Interestingly, the pathogenesis of late-onset psoriatic JIA resembles ERA with entheses inflammation and inflammation in the bowel wall [5]. Early-onset psoriatic JIA is characterised by adaptive immune mechanisms involvement with development of dactylitis [5].

Standing apart from non-systemic subtypes that are known as autoimmune disorders, sJIA has been suggested to be an autoinflammatory pathology with different pathogenesis [16]. In sJIA, uncontrolled activation of the innate immune system results in activation of monocytes/macrophages, neutrophils and immature (CD34 + CD33+) myelomonocytic precursors, and increased production of pro-inflammatory cytokines IL1β, IL6, IL18 and phagocyte-specific S100 proteins [16, 30, 31]. MAS is a complicated sJIA, where some triggers (bacterial or viral infections, drugs) cause uncontrolled expansion of cytotoxic CD8^+^ T-cells that produce pro-inflammatory cytokines and propagate the induction and activation of hemophagocytic macrophages that infiltrate bone marrow and multiple organs in particular the liver and spleen [8].

### Inflammatory cytokines

There is a significant and predominant pro-inflammatory cytokine signature in the plasma and synovial fluid of patients with JIA. JIA patients demonstrate high levels of TNFα, MIF, macrophage inflammatory protein (CCL3), macrophage-derived chemokine (CCL22), monokine induced by IFNγ (CXCL9), monocyte chemoattractant protein-1 (CCL2) and IFNγ-induced protein-10 (CXCL10) in blood and synovial fluid [32]. Comparison of patients with different subtypes showed significantly higher concentrations of plasma CCL11, CXCL10 and CCL2 in oligoarticular JIA compared to sJIA, while patients with sJIA demonstrated higher level of IL1, IL6 and IL18 in serum [32, 33]. Elevated levels of IL33 are observed in patients with RF+ polyarticular JIA in comparison with oligo- and RF+ polyarticular JIA, and are correlated with disease activity, indicative of being a potential biomarker candidate for pJIA disease activity [34]. The concentrations of MIF, IL10 and IL17 in serum or synovial fluid is predictive for oligoarticular JIA (with less than 60% accuracy). MIF, IL17 and IL23 are also increased in ERA [18]. IL18 is predictive for sJIA (with 93% accuracy) [32], and plays a pivotal role in the pathogenesis of MAS, with an increased concentration reported to be predictive of MAS complication in sJIA patients [35]. Martini et al. (2012) showed the correlation between elevated levels of pro-inflammatory cytokine IL6 in sJIA with the severity of joint inflammation and microcytic anaemia due to IL6 influence on erythropoiesis by increasing the synthesis of iron-lowering hormone hepcidin [6].

Leucine-rich *α*2-glycoprotein (LRG), induced by pro-inflammatory cytokines IL1, IL6 and TNFα, promotes differentiation and proliferation of Th17 cells and is present in the sJIA and MAS correlated with serum CRP and ferritin levels [36]. Adenosine deaminase-2 (ADA2), released by monocytes and macrophages following stimulation with IL18 and IFNγ is considered to be a novel biomarker of MAS, being strongly correlated with ferritin, IL18 and CXCL9 [37].

Anti-inflammatory cytokines are also involved in the progression of JIA [16]. The pleotropic effects of both transforming growth factor-beta (TGFβ) and IL10 impact on the control of innate and adaptive immunity. Thus, TGFβ1 directly targets T-cells leading to the established immune tolerance to self- and environmental antigens. IL10 mediates anti-inflammatory actions by induction of heme oxygenase-1, a stress-inducible protein with anti-inflammatory properties, and through the induction of mammalian target of rapamycin (mTOR) inhibitor [38]. Thus, the clinically inactive disease, in the absence of medication in some patients, may represent compensation of the autoimmune activity by anti-inflammatory cytokines [16]. Another anti-inflammatory cytokine, IL37 was found to be significantly elevated in plasma and IL-37 mRNA expression has been shown to correlate with disease activity and production of pro-inflammatory cytokines IL6, TNFα and IL17 [39].

The collective data available to date, suggests that cytokine patterns may be appropriate for accurate disease classification in early JIA with the potential as targets for improving diagnosis and treatment strategies for patients with paediatric autoimmune disease [33].

### Autoantibody production

Serological biomarkers in JIA patient tissues may be stratified into those that are stable and persistent throughout disease course (including antibodies such as RF) and those that change over time and disease activity (including cytokines such as IL18). ANA, RF, anti-cyclic citrullinated peptide (anti-CCP) and antibodies against mutated citrullinated vimentin (anti-MCV) are reported in non-systemic JIA pathogenesis [40, 41]. RF is an antibody specific to the Fc portion of IgG; it was first described as a key serological marker in patients with adult rheumatoid arthritis (RA) and then identified in a small sub-group of pJIA patients (only 5% of total JIA patients) [4]. RF-positivity is associated with severe prognosis of JIA and rapid formation of bone erosions [40]. ANA is considered to be common for oligoarticular, polyarticular, psoriatic subtypes of JIA, and associated with increase the risk of uveitis in JIA patients [42]. Anti-CCP and anti-MCV typically characterise RF-positive pJIA and may predict more severe and erosive disease progression needing earlier and more intensive therapy [40, 43]. Anti-CCP activates complement and macrophages by crosslinking TLR4 and Fc gamma receptors and inducing TNFα production by binding to the macrophage Fcγ receptor IIa in vitro*.* Patients double positive for Anti-CCP and RF have higher levels of TNFα, IL1β, IL6 and IL17 [41].

## Establishing diagnosis and prediction of complications

Clinical symptoms, family history, laboratory markers and instrumental examinations (ultrasound and magnetic resonance imaging) are used to determine JIA subtype. Physical examination findings are paramount and include signs of arthritis (pain, tenderness, stiffness and swelling of synovial joints) and extra-articular findings (such as rash, lymphadenopathy, dactylitis, nail changes). Laboratory tests for HLA-B27, RF or anti-CCP antibody identifies the subtype of JIA and the risk of bone erosions and joint damage. Myeloid-related protein (MRP)8, MRP14 and IL18 may be used as biomarkers for active sJIA, whereas HLA-B27 is predictive of ERA [41]. ANA and RF are useful for the diagnosis of oligo and pJIA subtypes [41]. ANA is associated with increased risk of chronic non-granulomatous uveitis, which is the most common extra-articular manifestation of JIA and is typically asymptomatic but has an elevated risk of causing visual impairment. Aljaberi et al. (2020) reported higher levels of pro-inflammatory calcium-binding S100 proteins in sJIA patients compared to other autoinflammatory syndromes. However, other studies have revealed that high baseline S100A12 concentration is associated with higher disease activity and response to methotrexate (MTX) and anti-TNF therapy in patients with JIA including pJIA, ERA, oligoarticular and psoriatic arthritis [44]. Thus, S100A8/9 and S100A12 proteins are subclinical inflammation markers that may help with diagnosis and monitoring disease activity [45].

Recent history of gastrointestinal or urinary infection, gut inflammation confirmed by elevated fecal calprotectin levels, sacroiliitis with inflammatory spinal changes and enthesitis detected by MRI support diagnosis of ERA [18]. Subclinical gut inflammation has also been identified in older-onset of psoriatic JIA [5].

The diagnosis of sJIA in accordance with ILAR criteria requires arthritis and fever within the last 2 weeks, and one of the following criteria: rash, generalised lymphadenopathy, enlargement of liver or spleen, or serositis [46]. Common laboratory abnormalities suggestive of systemic inflammation include elevated erythrocyte sedimentation rate (ESR), C-reactive protein (CRP), white blood cell count, platelet count, ferritin, transaminases, aldolase and d-dimers help to define the activity of the disease [47]. Laboratorial analysis of patients with active sJIA may reveal granulocytosis, thrombocytosis, anaemia, upregulation of acute phase reactants (elevated erythrocyte sedimentation rate (ESR) and C-reactive protein (CRP), which are typical findings but not so essential for diagnosis in comparison with the life-threatening complication of MAS that include pancytopaenia, increased levels of ferritin, liver enzymes (aspartate and alanine transaminases), triglycerides, d-Dimers and hypofibrinogenemia [7]. Clinical findings of MAS include high non-remitting fever, generalised lymphadenopathy, hepatosplenomegaly, central nervous system dysfunction and hemorrhagic manifestations.

## Treatment

Therapeutic intervention begins at diagnosis with non-steroidal anti-inflammatory drugs (NSAIDs) followed by disease-modifying anti-rheumatic drugs (DMARDs, most often methotrexate) and/or corticosteroid intra-articular injection. In blocking prostaglandin production via inhibition of cyclooxygenase-1 and cyclooxygenase-2, NSAIDs obtain both analgesic and anti-inflammatory effects. Local corticosteroid joint injections are effective in synovitis and may be a first-line treatment for oligoarthritis alone or in addition to DMARDs. Systemic administration of high dose corticosteroids provides good short-term effect, especially in sJIA patients, but has no influence on the long-term disease outcome. Moreover, its prolonged administration is associated with severe side effects including osteoporosis, growth suppression, immunosuppression and metabolic effects (Table 4) (48).

**Table 4:** The mechanism of action and side effects of commonly used medications for JIA treatment. DMARDs - disease-modifying anti-rheumatic drugs, GC – glucocorticoids, ERA - enthesitis-related arthritis, IL - interleukin, NK - natural killer, MAS – macrophage activation syndrome, MTX – Methotrexate, sJIA – systemic JIA, TNF - tumor necrosis factor

Drug	Mechanism of Action	Therapeutic Options	Adverse Event	Reference
**Non-Biologic DMARDs**				
MTX	• MTX is a structural analogue of folic acid that inhibits dihydrofolate reductase and DNA synthesis• Acts in different pathway: cytokine production, arachidonic acid metabolism and cell apoptosis	• Polyarticular JIA• Oligoarticular JIA• JIA-related uveitis refractory to topical treatment• sJIA with predominant joint inflammation and without active systemic symptoms• Psoriatic JIA	• Nausea• Oral ulceration• Infections (herpes zoster)• Severe complications in less than 1% of cases include:- Cirrhosis- Pneumonitis- Leucopenia- Thrombocytopenia- Anaemia	[49–51, 53, 54]
Leflunomide	• Inhibition of T-cell proliferation by blocking pyrimidine synthesis	• Polyarticular JIA patients who cannot tolerate MTX• Used rarely in pediatric patients because of its teratogenicity and long half-life	• Diarrhoea• Rashes• Cytopenia• Abnormal liver-function test• Teratogenicity	[52]
Sulfasalazine	• Immune-suppressive effect not fully established	• ERA with moderate activity, but not in other types of JIA	• Gastrointestinal toxicity• Sulphonamide allergy• Neuropsychiatric complications (headache, anxiety)• Pancytopenia• Pneumonitis• Myelosuppression• Hypogammaglobulinaemia	[18, 54]
**Biologic DMARDs**				
TNF inhibitors				
Adalimumab	• Subcutaneous recombinant human IgG1κ monoclonal antibody• Neutralises TNFα by binding with soluble and membrane-bound TNF	• JIA patients with resistance or intolerance to MTX• Polyarticular JIA• JIA with uveitis• ERA refractory to sulfasalazine• Psoriatic JIA	• Risk of reactivation of latent infections such as tuberculosis, and new infections caused by viruses, fungi, or bacteria• Rare reports of:- Lymphoma- Demyelinating central nervous system disorders- Cardiac failure	[58]
Infliximab	• Intravenous chimeric monoclonal antibody against TNFα• Binding with soluble and transmembrane TNFα, that mediates complement and antibody-dependent cytotoxicity of expressed TNFα cells (macrophages and monocytes)	• Polyarticular JIA where there has been the use of MTX for at least 3 months with poor response• Uveitis• Psoriatic JIA	• Opportunistic infections: herpes, tuberculosis, pseudomonas pneumonia, reactivation of hepatitis B, fungal infection	[56, 57]
Etanercept	• Fusion protein consisting of the extracellular domain of the human p75 TNFα receptor• Linked to the Fc region of human IgG1, binds and inhibits soluble TNFα	• Polyarticular JIA with resistance or intolerance to MTX• ERA• Psoriatic JIA	• Central nervous system events (headache, neuritis)• Varicella infections• Rare:- Malignancy	[54–56]
IL1 inhibitors				
Anakinra	• Recombinant IL1 receptor antagonist binds to IL1 receptors (IL1r1)• Inhibits the binding of IL1α and IL1β	• Refractory sJIA with persistent systemic symptoms• MAS	- Vomiting, nausea, diarrhea- Headache- Abdominal pain- Upper respiratory and urinary tract infections- Neutropenia	[61, 63]
Canakinumab	• Human Monoclonal antibody• Selectively blocks IL1β	• sJIA patients with continued disease activity after treatment with GC monotherapy and MTX or leflunomide, anakinra or tocilizumab	• Thrombocytopenia• Neutropenia• Upper respiratory tract infection• Cough• Abdominal pain• Gastroenteritis, vomiting, diarrhea• Pyrexia• Very rare:- pneumococcal sepsis	[64]
Rilonacept	• Fusion protein between the Fc portion of IgG and the IL1 receptor• Blocks the interaction of IL1 with cell surface receptors preventing IL1 signalling	• Active sJIA	• Infections• Developed elevations in liver transaminases• High cholesterol or triglycerides• Abdominal pain• Gastroenteritis, nausea, diarrhea	[67]
T-cell inhibitors				
Abatacept	• Inhibitor of naïve T-cell activation• Soluble fusion protein of CTLA-4 with the Fc portion of IgG that binds to CD80/CD86 and blockades signal following MHC-peptide: TCR engagement necessary for T cell activation	• Severe sJIA• Polyarticular JIA patients with inadequate response to MTX and TNF-blockers	• Bacterial and opportunistic infections• Rare:- acute lymphoblastic leukemia	[54, 59]
IL6 inhibitors				
Tocilizumab	• Humanised monoclonal antibody against the IL6 ubiquitous receptor (IL-6R)• Block IL6 signaling pathway by binding to cell-surface and soluble IL-6R	• sJIA• Polyarticular JIA with resistance and continued disease activity after treatment with MTX and TNF-blockers	• Headache• Upper respiratory tract infections (more than 10%)• Varicella, herpes zoster• Neutropenia• Elevation of aminotransferases	[30, 31]
Anti-B-cells therapy				
Rituximab	• Chimeric monoclonal antibody against B-cells with mouse variable and human constant regions• Binds CD20 on the surface of B-cells forming a cap that allow NK cells to destroy B cells• Leads to B-cell death and removal from circulation	• JIA refractory to anti-TNF agents and standard immunosuppressive therapy	• Infusion reactions (headache, throat irritation, rash, itchiness, pyrexia) in one third of patients• Bacterial infections• Hepatitis B reactivation• Rare:- cardiac arrest- cytokine release syndrome- multifocal leukoencephalopathy- pulmonary toxicity	[54, 70]
**Janus Kinase (JAK) inhibitors**				
Tofacitinib	• Inhibit JAK1 and JAK3• Interrupt the JAK-STAT signalling pathway, which is responsible for the transmission of extracellular multiple proinflammatory cytokines, including IL-6, into the nucleus, leading to changes in DNA transcriptome	• Refractory polyarticular JIA• sJIA refractory to other therapy	• Diarrhea• Headache• High blood pressure• Upper respiratory tract infections• Varicella zoster virus reactivation• Cytomegalovirus infection• Pulmonary embolism• Rare:- Lymphoma or other malignancies	[74]
**Other medications**				
Glucocorticoids (GC)	• Binding to glucocorticoid receptors inhibits calcium and sodium cycle across plasma membranes, reducing activation and proliferation of immune cells• Post-transciptional destabilisation of messenger RNA resulting in reduced production of proinflammatory cytokines including IL1 and IL6	√ Systemic steroids for:• sJIA with serious organ involvement (including pericarditis, myocarditis)• Patients with features indicative of MAS• High disease activity in oligo- and polyarticular JIA• Intraarticular steroids for oligo- and polyarticular JIA	• Infections• Myopathy• Neuropsychiatric symptoms• Osteoporosis• Obesity• Insulin resistance• Cushing syndrome• Gastric ulcer• Cataract• Glaucoma	[48]
Cyclosporine A	• A fungal cyclic polypeptide• Binds to the cellular protein cytophilin, resulting in inhibition of the enzyme calcineurin• Specifically and reversibly inhibits CD4+ immunocompetent lymphocytes in the G0-G1 phase of the cell cycle• Then inhibits IL2 production and release by T-helpers	• sJIA with indication of MAS	• Nausea• Headache• Renal complications• Neuronal complications (paresthesia),• Hepatotoxiety	[48]

The American College of Rheumatology (ACR) recommends early use of DMARDs, specifically MTX, leflunomide and/or sulfasalazine (Table 4) [48]. MTX is considered to be the first choice DMARD for oligo- and pJIA when NSAIDs and intraarticular steroids are insufficient [49–51]. MTX is also considered to be effective in children with PsJIA, though the axial manifestations limits prescription of MTX and so TNF inhibitors are typically required in these cases [5]. Leflunomide may be used as an alternative DMARD for pJIA in cases of MTX intolerance [52, 53]. Sulfasalazine is recommended for patients with moderate activity of ERA with active peripheral arthritis, but is inefficient in case of sacroiliitis [18, 54]. The Clinical Commissioning Policy Statement: Biologic Therapies for the treatment of Juvenile Idiopathic Arthritis (2015) reports that in 30–50% of patients where disease continues to progress, advanced biologics are the next therapeutic step.

The first biological drugs registered for the treatment of JIA were anti-TNFα agents, etanercept and adalimumab. Etanercept was approved for the treatment of pJIA in 1999, based on a randomised, placebo-controlled double-blind study evaluation of safety and efficacy [55]. Now TNFα inhibitors are recognized to be the most effective drugs for the treatment of JIA with influence on pain, stiffness, growth and quality of life and were first successful in the treatment of pJIA, then ERA, psoriatic and oligoarthritis subtypes [48, 56, 57]. Combination of TNFα blocking agents with MTX increases the opportunity of achieving JIA remission in patients with these subtypes and is an effective option in uveitis-associated JIA [58]. In a randomized double-blind trial anti-TNF agents namely etanercept or adalimumab have proven effective for ERA [18].

For pJIA that is nonresponsive to at least one DMARD, including TNFα inhibitors, abatacept (CTLA4-Ig) may be recommended [54, 59] following demonstration of long-term efficacy, safety and improvement of quality of life in 58 JIA patients for 7 years [59]. Another option should TNFα inhibition reach sub-optimal clinical outcomes for pJIA is the IL6 receptor inhibitor, tocilizumab. Tocilizumab might also be a treatment option for JIA-related uveitis refractory to MTX and TNF inhibitors [60].

For many years anti-TNFα therapy demonstrated improved treatment outcomes for all forms of JIA but were less effective for sJIA, where the therapeutic approach has been IL1β/IL6 signalling blockade [56, 61–63]. Tocilizumab (anti-IL6R) was the first approved medication for the treatment of active sJIA, demonstrating safety and efficacy in two multicentre studies of patients with sJIA and pJIA [31]. Other studies have shown the efficacy of IL1 blockade in sJIA [61]. Complete remission was obtained in seven out of nine patients with refractory sJIA treated with recombinant IL1 receptor antagonist anakinra (the other two patients demonstrating a partial treatment response) [62]. However, Quartier et al. (2011) reported a short-term effect of this drug in corticosteroid-dependent patients with sJIA in a randomised, double-blind, placebo-controlled trial and showed that anakinra is less effective on arthritis than on systemic symptoms [63]. Currently, anakinra (IL1Ra), rilonacept (IL1 inhibitor) and canakinumab (anti-IL1β) have been successfully studied in clinical research with comparable long-term efficacy where half of treated patients achieved remission [61, 64–67]. IL18 may be another target for treatment of sJIA resistant to IL1 and IL6 inhibition, as far as higher levels of IL18 have been associated with high ferritin levels reported in MAS [68, 69]. 

MAS as a form of hemophagocytic lymphohistiocytosis is usually treated with high-dose methylprednisolone and cyclosporine A (a calcineurin inhibitor). In relation to biological therapies, treatment of MAS has been successful using IL1 receptor antagonist anakinra (IL-1Ra) and rituximab (anti-CD20) [70], which has also demonstrated efficacy in other immunological disorders, including SLE [61]. Other promising therapeutics, tadekinig alfa (anti-IL18) and emapalumab (anti-IFNγ) are currently undergoing clinical trials with data reporting safety and potential efficacy for the treatment of sJIA and MAS [71, 72].Another new class of biological DMARDs are the Janus**-**associated tyrosine kinases (JAK) inhibitors. The mechanism of their action consists of blocking JAK-STAT pathways to interrupt the transduction of extracellular pro-inflammatory signals into the cell nucleus. The efficacy of first generation of JAK inhibitors (namely tofacitinib and baricitinib) were first explored in adults with RA, and then in other immune-mediated inflammatory diseases such as ankylosing spondylitis, SLE, inflammatory bowel disease and psoriasis [73]. Tofacitinib was effectively used in case of refractory sJIA [74]. Miserocchi et al. (2020) showed effectiveness of tofacitinib and baricitinib in 4 cases of JIA uveitis, defined by a reduction of intraocular inflammation according to Standardized Uveitis Nomenclature criteria, and no side effects were registered [75]. The results of a randomised phase 3, multinational, double-blind, controlled clinical trial (NCT02592434) has proven the safety and effectiveness of tofacitinib in pJIA, resulting in reduced flares and disease activity [76]. Ongoing trials are investigating baricitinib (NCT04088396, NCT03773978, NCT04088409) and tofacitinib (NCT03000439) in patients with other JIA disorders [77].Recent therapeutic advances including combination of DMARDs, corticosteroids and the biological agents reduce synovitis, tissue damage progression and systemic complications, making low disease activity an achievable goal in JIA. Early treatment with biologics may be important in controlling disease activity and avoiding steroids altogether or at least reducing the duration of use. However, long-term prescription of biologic agents due to immune suppression increases the risk of opportunistic infections and potentially even malignancy (the main adverse events are shown in the Table 4) [56]. Early aggressive treatment of JIA was shown to be beneficial with 40% of patients achieving clinical inactive disease within 6 months [78], though some serious adverse events was registered during treatment (such as pneumonia, septic joint, elevation of transaminases, peritonsillar abscess, recurrent herpes simplex). It may be suggested that some of these patients would have achieved remission even with less aggressive treatment [79].

## Disease course, quality of life and functional outcome

Substantial progress in JIA treatment has been made over the last three decades. Clinical outcomes have dramatically improved, with disease control and remission possible in most patients. Nevertheless, a significant proportion of patients have ongoing disease activity. In fact, about half of patients continue to require active treatment into adult life, whereas complete remission is achieved in only 20–25% of patients [80, 81].

Administration of biological agents has decreased the mortality rate of JIA from 1 to 4% in 1970s to 0.3–1% in 2016 [82]. Improved clinical outcomes in physical disability are reflected in the Steinbrocker functional classification scale. Between 1976 and 1994, 15% of JIA patients were within Class III (limited to few or no activities of the patient’s usual occupation) and Class IV (bedridden with little or no self-care), compared to 5% in 2002 [83]. However, joint damage, occurring before treatment led to surgical intervention in 14% of patients, emphasising the importance of early aggressive treatment to achieve complete remission [84]. The prominent factor influencing treatment outcome is presence of systemic manifestation. Multi-organ failure in patients with MAS is fatal in approximately 8% of cases [8].

Being the most frequent extraarticular manifestation JIA**-**associated uveitis became the main cause of vision loss in childhood, and furthermore about half of these patients suffer from active uveitis in adulthood [85]. Additionally, a high risk of osteoporosis and consequently fractures in early adulthood remain higher in JIA patients even in remission [15].

Long-term outcomes of JIA patients are dependent on subtype and disease activity, which may remain elevated for many years including into adulthood. In early adulthood about half of patients with JIA have active disease and approximately 30% suffer from some form of disability [80]. Selvaag et al. (2016) reported a 59% remission rate in patients with JIA after 30 years but noted the low quality of life in adults with JIA [81].

Comorbidities and complications highlight the status of JIA as the most important paediatric rheumatological pathology that may continue with remissions and flares throughout life, leading to impairment of connective tissue and the reduction of life quality. There is a need for more discovery science research to help the understanding of the complexity of the inflammatory process and to enable the development of treatments that may come with the promise of an actual cure.

## Conclusions

JIA is a chronic rheumatic disease of childhood, characterised by progressive joint destruction and serious systemic manifestations. Complex interactions between immune cell populations, including lymphocytes, monocytes, macrophages and neutrophils, trigger the pathophysiological cascade in JIA. Our review of clinical research has demonstrated that the heterogeneity of non-systemic and sJIA pathogenesis stratifies JIA patients by subtype, with requirement for differing therapeutic approaches. A broad range of DMARDs such as T-cell inhibitors, anti-TNFα agents, IL1 and IL6 blockers, JAK inhibitors have significantly improved the clinical management of JIA. However, further research is needed to deepen our understanding of the complexity of the inflammatory process in JIA and to enable the development of effective treatments that improve upon clinical outcomes and disease remission.

## Data Availability

Not applicable.

## Abbreviations

JIA: Juvenile idiopathic arthritis
ILAR: International League of Associations for Rheumatology
RF: Rheumatoid factor
sJIA: Systemic juvenile idiopathic arthritis
ERA: Enthesitis-related arthritis
pJIA: Polyarticular JIA
PRINTO: Pediatric Rheumatology International Trials Organization
Early-onset ANA+ JIA: Early-onset antinuclear antibody–positive juvenile idiopathic arthritis
AOSD: Adult-onset Still’s disease
MAS: Macrophage activation syndrome
ANA: Antinuclear antibodies
RF+ JIA: Rheumatoid factor–positive juvenile idiopathic arthritis
Anti-CCP: Anti-cyclic citrullinated peptide
CRP: C-reactive protein
HLA: Human leukocyte antigen system
VEGF: Vascular endothelial growth factor
EC: Endothelial cell
sVEGF-R: Soluble vascular endothelial growth factor receptor
OPN: Osteopontin
Ang-1: Angiopoietin-1
TNF: Tumour necrosis factor
IL: Interleukin
MMPs: Matrix metalloproteinases
RANK: Receptor activator of nuclear factor-kappaB
MIF: Macrophage migration inhibitory factor
PTPN22: Protein tyrosine phosphatase non-receptor type-22
STAT4: Signal transducer and activator of transcription-4
SLC11A1: Solute carrier family-11 member-1
NRAMP1: Natural resistance-associated macrophage protein-1
WISP3: WNT1-inducible signalling pathway protein-3
ERAP: endoplasmic reticulum resident aminopeptidases
NK cells: Natural killer cells
DC: Dendritic cells
Anti-MCV: Antibodies against mutated citrullinated vimentin
Anti-CCP: Anti-cyclic citrullinated peptide
Th: T helpers
Treg: Regulatory T-cells
CCL2: C-C motif chemokine ligand-2; Monocyte chemoattractant protein-1
CCL3: C-C motif chemokine ligand 3; Macrophage inflammatory protein-1α
CCL22: C-C motif chemokine ligand-22; Macrophage-derived chemokine
CXCL9: C-X-C motif chemokine ligand-9; Monokine induced by IFNγ
CXCL10: C-X-C motif chemokine ligand-10; IFNγ-induced protein 10
LRG: leucine-rich α2-glycoprotein
ADA2: adenosine deaminase-2
TGF: Transforming growth factor
mTOR: Mammalian target of rapamycin
RA: Rheumatoid arthritis
MRP: Myeloid-related protein
ESR: Erythrocyte sedimentation rate
CRP: C-reactive protein
NSAIDs: Non-steroidal anti-inflammatory drugs
DMARDs: Disease modifying anti-rheumatic drugs
MTX: Methotrexate
ACR: American College of Rheumatology
GC: Glucocorticoids
